# Cerium Oxide-Tungsten Oxide Core-Shell Nanowire-Based Microsensors Sensitive to Acetone

**DOI:** 10.3390/bios8040116

**Published:** 2018-11-23

**Authors:** Milena Tomić, Milena Šetka, Ondřej Chmela, Isabel Gràcia, Eduard Figueras, Carles Cané, Stella Vallejos

**Affiliations:** 1 Instituto de Microelectrónica de Barcelona (IMB-CNM, CSIC), Campus UAB, 08193 Bellaterra, Spain; milena.tomic@imb-cnm.csic.es (M.T.); isabel.gracia@imb-cnm.csic.es (I.G.); Eduard.Figueras@imb-cnm.csic.es (E.F.); carles.cane@imb-cnm.csic.es (C.C.); 2 CEITEC-Central European Institute of Technology, Brno University of Technology, 61200 Brno, Czech Republic; milena.setka@ceitec.vutbr.cz (M.Š.); Ondrej.Chmela@ceitec.vutbr.cz (O.C.)

**Keywords:** gas sensors, volatile organic compounds (VOCs), acetone, metal oxides, heterojunctions

## Abstract

Gas sensitive cerium oxide-tungsten oxide core-shell nanowires are synthesized and integrated directly into micromachined platforms via aerosol assisted chemical vapor deposition. Tests to various volatile organic compounds (acetone, ethanol, and toluene) involved in early disease diagnosis demonstrate enhanced sensitivity to acetone for the core-shell structures in contrast to the non-modified materials (i.e., only tungsten oxide or cerium oxide). This is attributed to the high density of oxygen vacancy defects at the shell, as well as the formation of heterojunctions at the core-shell interface, which provide the modified nanowires with ‘extra’ chemical and electronic sensitization as compared to the non-modified materials.

## 1. Introduction

Odor (gas, vapor, aroma) detection systems are of high interest as they are non-invasive key-enabling technologies, which are relevant in both traditional (e.g., environment, industry) and innovative applications such as the early detection of diseases from exhaled breath [1,2]. The literature related to exhaled breath as bio information for disease diagnosis has shown previously that human breath contains more than two hundred different gases and volatile organic compounds (VOCs) species that vary from person to person [3]. In the midst of a wide range of analytes, acetone, toluene, and ethanol are within the most relevant VOCs that are typically involved in various diseases, including diabetes and cancer. Thus, for instance, high acetone concentrations (~21 ppm) have been identified in the exhaled breath of diabetic patients, as compared to healthy patients (~2.7 ppm) and oppositely to patients with lung cancer, who showed lower acetone concentrations (~0.9 ppm). Similarly, the ethanol concentration in breath has shown an increase (~2.1 ppm) in patients with lung/breast cancer and diabetes with respect to healthy patients (~0.2 ppm) [4,5,6].

Currently, the analysis of breath is still an emerging diagnosis technology that uses large and expensive laboratory equipment such as gas-chromatograph, ion-mobility and/or mass spectrometers [7]. In the future, however, miniaturized, portable, and wearable systems with enhanced functionality (sensitivity, selectivity, and stability) and high autonomy at a low cost could substitute this equipment; a fact that demands the innovation of current odor detection systems. In this context, metal oxide (MOX) gas sensors based on the chemoresistive principle represent an alternative to bulky equipment providing simpler architecture and fabrication processes compatible with ‘standard’ MEMS and CMOS technologies [2]. Chemoresistive gas sensors devices generally consist of a transducing platform (microscale) and a gas sensitive MOX optimized to interact with specific groups of gaseous or vapor analytes. However, among the diverse issues that may potentially be addressed to improve the functionality of these monitoring systems (e.g., optimization of sensing modes and control electronics or the integration of smart systems with new micro/nano fabrication concepts), the focus on nanoscaled sensitive materials is still essential to radically improve their performance. Thus, several studies have demonstrated that MOXs modified with second-phase constituents, either nanosized noble metals or other MOXs, have a positive effect on the sensing properties of both the host MOX and the second-phase constituent, particularly when the size of both materials is within the Debye length of the surface (typically on the order of 2−100 nm) [8]. Moreover, recently, it has been pointed out that the modification of a MOX with noble metals or other MOXs allows for the formation of nanoscale heterojunctions and, in turn, sensing mechanisms dominated not only by the surface, but also the interface, which has proved to improve the sensing properties of these materials [9]. 

**Figure 1:**
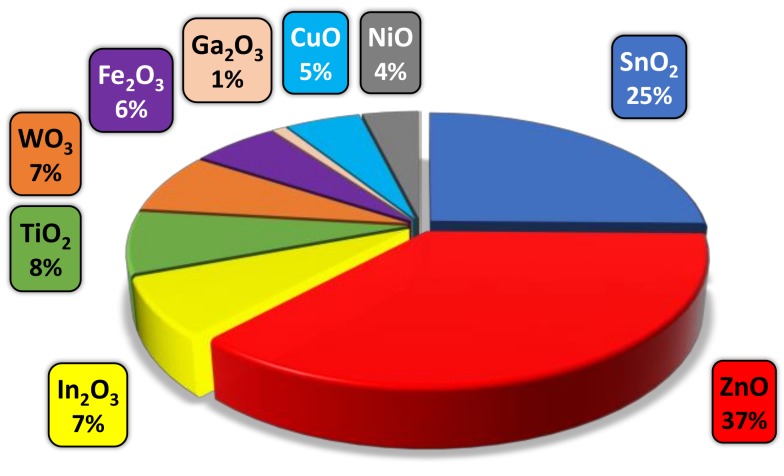
A survey of the most applicable gas sensitive MOXs reported in the literature (Web of Science database from 1998 to 2018).

In recent years, tungsten oxide has demonstrated high potential in gas sensing among traditional gas sensitive MOXs such as SnO_2_ and ZnO (Figure 1), showing a strong sensitivity to oxidizing gases including nitrogen dioxide and ozone [10]. Moreover, the modification of tungsten oxide with second-phase constituents such us platinum, copper oxide, or iron oxide has shown an improved sensitivity and selectivity to reducing species including hydrogen [11], hydrogen sulfide [12] or toluene [13], respectively. As far as cerium oxide is concerned, the peculiarity that makes this oxide also attractive in gas sensors reside overall in the defect sites caused by the valence state changes between Ce^4+^ and Ce^3+^, which considerably alter the concentration of oxygen vacancies, and provides a good redox behavior and catalytic activity [14,15,16]. However, and despite these favorable surface properties the use of cerium oxide in gas sensing is still infrequent (Figure 1) and its sensing properties upon VOCs have not been fully explored in the literature related to gas sensors.

Optimized gas sensitive MOXs need synthetic methods able to produce well defined and even structures. Additive (bottom-up) synthetic methods, as opposed to subtractive (top-down) methods, are ideal for this task and industrially attractive as they provide the ability to generate films in a continuous mode with high purities and high throughput. Aerosol assisted (AA) chemical vapor deposition (CVD) is a versatile additive synthetic method used previously to obtain non-modified (e.g., WO_3_) or metal/MOX modified MOXs (e.g., Pt/WO_3_, Fe_2_O_3_/WO_3_) [11,13]. Additionally, recently, the AACVD of cerium oxide from Ce (dbm)_4_ has been proved as a strategy to overcome the low volatility of traditional cerium CVD precursors [17]. In this work, however, we achieve the AACVD of cerium oxide from Ce (acac)_3_ precursor and use this route to synthesize cerium oxide-tungsten oxide core-shell nanowires in a two-step process performed directly on silicon-based micromachined platforms. In addition, we validate the sensing properties of these systems to acetone, and other relevant VOCs monitored in early disease diagnosis. 

## 2. Materials and Methods

**Figure 2:**
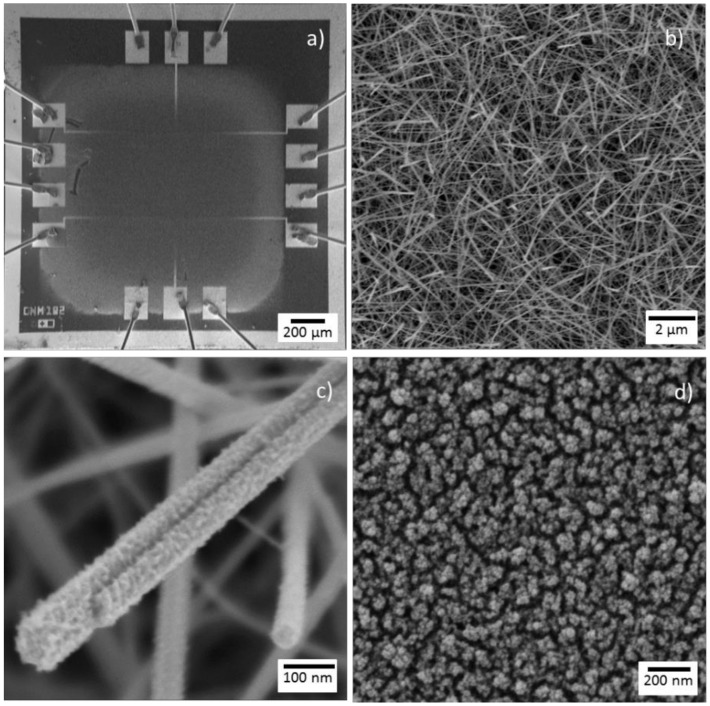
The SEM imaging of a (**a**) gas sensor device, the *Ce*/*W* nanowires at (**b**) low and (**c**) high magnification, and (**d**) the non-modified *Ce* films integrated on the micromachined membrane.

Tungsten oxide (non-modified nanowires), cerium oxide (non-modified porous films), and cerium oxide-tungsten oxide core-shell nanowires were grown directly onto micromachined transducing platforms (Figure 2a) [18] using the AACVD system described previously [19]. AACVD is a variant of the conventional CVD technique, which uses aerosol to transport dissolved precursors to a heated reaction zone. Here, the non-modified tungsten oxide nanowires films were deposited at 350 °C from a solution of tungsten hexacarbonyl (30 mg, W(CO)_6_, Sigma-Aldrich, St. Louis, MO, USA, ≥97%) and methanol (5 mL Sigma-Aldrich, ≥99.6%), whereas the non-modified cerium oxide films were deposited at 500 °C from cerium (III) acetylacetonate hydrate (28 mg, Ce (acac)_3_·×H_2_O, Sigma-Aldrich) dissolved in methanol (2 ml, Sigma-Aldrich). On the other hand, the cerium oxide-tungsten oxide core-shell nanowires were achieved using a two-step AACVD process [20], in which the tungsten oxide nanowire cores were deposited at 350 °C in the first step and the cerium oxide shell film at 500 °C in the second step employing the same protocols described above for the non-modified films. Finally, the non-modified and modified films were annealed at 500 °C in air. 

The morphology of the films was examined using scanning electron microscopy (SEM— Auriga Series, 3 KV, Carl Zeiss, Jena, Germany) and the phase using X-ray Diffraction (XRD–Bruker-AXS, model A25 D8 Discover, Cu Kα radiation, Billerica, MA, USA). Further analysis of the material was carried out using X-ray photoelectron spectroscopy (XPS—Kratos Axis Supra with monochromatic Al Kα X-ray radiation, an emission current of 15 mA and hybrid lens mode, Manchester, UK). The survey and detailed spectra were measured using pass energy of 80 eV and 20 eV, respectively. The band gap of the films was estimated by measuring the diffuse reflectance (AvaSpec-UV/VIS/NIR, Avantes, Apeldoorn, the Netherlands) of the films and performing Kubelka–Munk transformation. 

The microsensors were tested in a continuous flow test chamber provided with mass flow controllers that allow the mixture of dry/humid air and calibrated gaseous analytes (ethanol, acetone, toluene, carbon monoxide and hydrogen purchased from Praxair, Danbury, CT, USA) to obtain the desired concentration. To have a proper control of the relative humidity (RH) inside the gas test chamber, an evaluation kit (EK-H4, Sensirion AG, Stäfa, Switzerland) with a humidity sensor was also used. The dc resistance measurements of the microsensor were achieved in a system provided with an electrometer (Keathley 6517A, Cleveland, OH, USA) and a multimeter (Keathley 2700, Cleveland, OH, USA) with switch system to monitor various sensors simultaneously. More details of the characterization systems were reported elsewhere [18]. The sensor response was defined as Ra/Rg, where Ra and Rg are the resistance in dry/humid air and the resistance after 600 s of analyte exposure, respectively. The sensors were tested for a period of one month during which each sensor accumulated 180 h of operation under the different conditions (analytes, temperatures, humidity) employed.

## 3. Results

### 3.1. Gas Sensitive Films

SEM imaging of the microsensors after AACVD of the gas sensitive structures showed uniform deposited films that covered the electrodes integrated into the micromachined membrane (Figure 2a). A close view of the non-modified tungsten oxide wires (*W*) showed bare and even surfaces as noticed previously for other AACVD tungsten oxide structures [21]. In contrast, a close view of the cerium oxide-tungsten oxide core-shell wires (*Ce*/*W*) displayed the presence of a rugged thin film covering the wire surface (Figure 2b,c), similarly to that observed when depositing non-modified cerium-based films (*Ce*) from a Ce (acac)_3_ methanolic solution via AACVD (Figure 2d).

**Figure 3:**
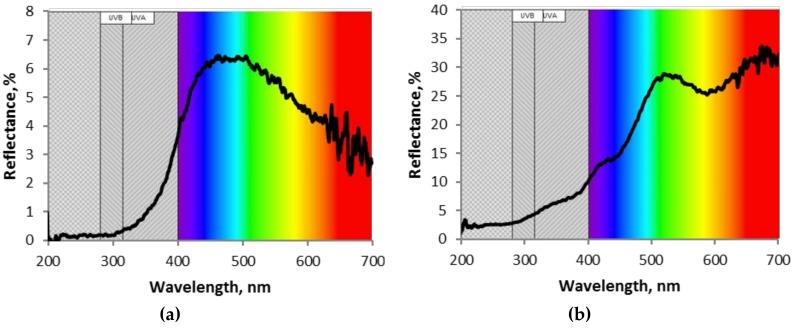
The diffuse reflectance spectra of the aerosol assisted chemical vapor deposited (**a**) tungsten oxide and (**b**) cerium oxide films without modification.

Generally, AACVD of the non-modified (*W* and *Ce*) and modified (*Ce*/*W*) films showed a good adherence to the substrate, with the wire-like morphology films (i.e., *W* and *Ce*/*W*) forming a mat-like network of non-aligned nanowires with diameters below 100 nm, and the particle-like morphology films (i.e., *Ce*) displaying a porous surface composed of grains with diameters below 40 nm. The as-deposited non-modified *W* films displayed a bluish color, whereas the *Ce*/*W* films displayed a dark yellowish to dark green color, similarly to the color observed on the *Ce* films. However, after annealing the non-modified *W* and modified *Ce*/*W* films became whitish and pale yellowish, respectively. Figure 3 displays the diffuse reflectance spectra of the films deposited without modification (i.e., only tungsten oxide or only cerium oxide) via AACVD. These measurements and their corresponding Kubelka–Munk transformation indicated optical band gaps at ~3.2 eV for the tungsten oxide films and ~3.1 eV for the cerium oxide films, in agreement with the literature band gap values of tungsten oxide (2.6–3.7 eV) [22] and cerium oxide (2.7–3.4 eV) [15]. 

**Figure 4:**
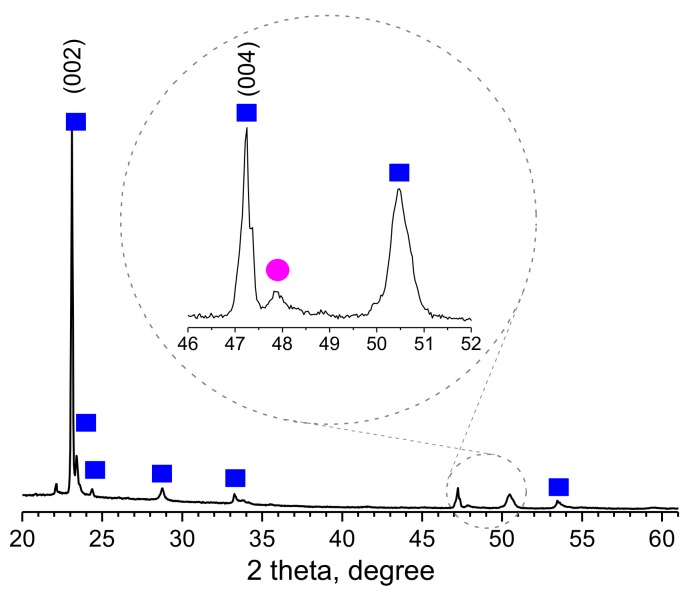
The XRD pattern of the *Ce*/*W* films. The diffraction peak at 47.8° 2θ (full pink circle) corresponds to cerium dioxide cubic phase (P1), COD ID card no. 7217887, the rest of the diffraction peaks (full blue squares) in the data can be indexed to a monoclinic phase (P21/n), ICDD card no. 72-0677, with only peaks of greatly enhanced intensity (preferred orientation), specifically indexed.

XRD analysis of the films revealed the presence of a monoclinic-phase (International Centre of Diffraction Data–ICDD card no. 72-0677) in the *W* and *Ce*/*W* films with greatly enhanced intensity (preferred orientation) in the [001] direction, consistent with our previous results for AACVD of tungsten oxide [13]. A weak diffraction peak was also noticed at 47.8° 2θ for the *Ce*/*W* films (Figure 4). This diffraction peak is in line with the pattern identified on the non-modified *Ce* based films corresponding to cerium dioxide (Crystallography Open Database–COD ID card no. 7217887).

**Figure 5:**
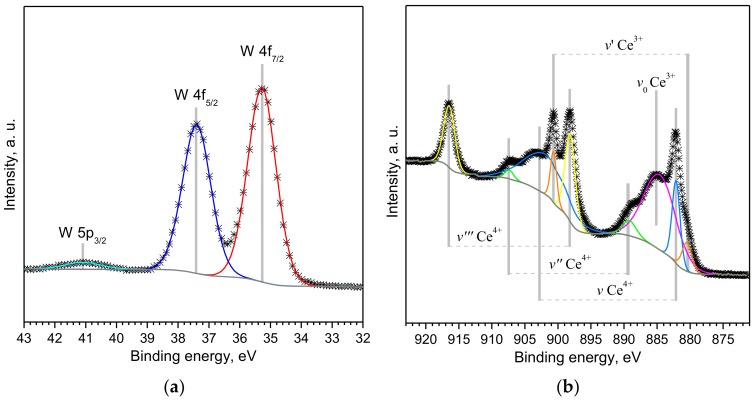
(**a**) The W 4f and (**b**) Ce 3d spectra recorded on the cerium-modified wires. The W 4f spectrum recorded on the non-modified tungsten oxide wires showed similar characteristics.

The XPS of both *W* and *Ce*/*W* films exhibited typical W 4f_7/2_, W 4f_5/2_ and W 5p_3/2_ XPS core level peaks (Figure 5a), consistent with the literature and previous tungsten oxide nanowires synthetized via AACVD [13]. XPS narrow scan spectra of the Ce 3d core level peaks at the *Ce* and *Ce*/*W* wires displayed multiplet splitting between 875 and 920 eV in agreement with the standard binding energies for Ce 3d peaks and partially reduced cerium oxide [23,24]. Figure 5b displays the experimental data and the corresponding deconvolution of the Ce 3d spectrum recorded on the *Ce*/*W* wires. The peaks *v, v’’* and *v’’’* are attributed to the main and satellite peaks of the Ce^4+^ state, whereas the peaks *v*_0_, *v*’ correspond to the peaks of Ce^3+^ state. The relative contribution of Ce^4+^ and Ce^3+^ species at the *Ce*/*W* films was estimated from the ratio of integrated Ce^4+^ peaks to the total Ce^4+^ and Ce^3+^ peaks, finding a value of ~42% for Ce^4+^ and 58% Ce^3+^ species. The relatively high amount of Ce^3+^ species indicate a charge imbalance with oxygen vacancy defects and an unsaturated chemical bond at the *Ce*/*W* film suggesting a high redox nature of the film. 

**Figure 6:**
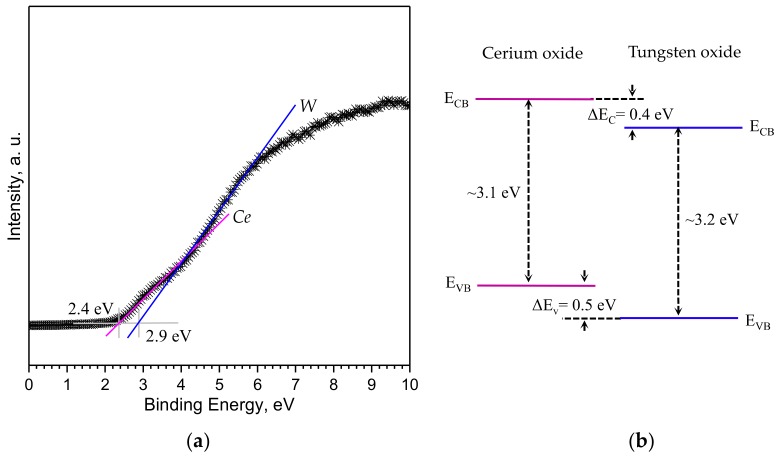
(**a**) The XPS valence band spectra of the cerium-modified tungsten oxide nanowires, and (**b**) schematic of the estimated energy level diagram at the interface. E_CB_ and E_VB_ represent the conduction band minimum and the valence band maximum (not to scale).

Linear extrapolation of the valence band (VB) leading edge on the XPS spectra recorded on the *Ce*/*W* film near the Fermi level (E_B_ = 0) indicates the simultaneous presence of both cerium oxide and tungsten oxide induced VB (Figure 6a). One can notice that the VB onset for cerium oxide occurs ~0.5 eV (ΔE_V_) above the VB onset for tungsten oxide. Therefore, according to the band gap estimated by diffuse reflectance for each non-modified material in the position of the conduction band (CB) of cerium oxide is ~0.4 eV (ΔE_C_) above the CB of tungsten oxide, consequently suggesting a staggered type of heterojunction at the interface of the *Ce*/*W* core-shell structures (Figure 6b). In contrast, the linear extrapolation of VB leading edge on the XPS spectra of the *W* and *Ce* films showed only the presence of tungsten oxide induced VB at 2.9 eV in agreement with previous reports [13].

In brief, the characterization of the films deposited via AACVD demonstrates the formation of crystalline cerium oxide-tungsten oxide core-shell wires with a relatively high amount of Ce^3+^ species at the surface and the presence of the characteristic valence band onsets for cerium oxide and tungsten oxide. 

### 3.2. Gas Sensing Tests

Overall, the sensors displayed an n-type response with a good reproducibility to the target gaseous analytes (acetone, ethanol, toluene, carbon monoxide, and hydrogen) and relatively low variations of the baseline resistance along the testing period. Gas sensing tests of the microsensors were carried out at various operating temperatures from 150 °C to 400 °C by DC resistance measurements of the films integrated via AACVD. These test proved a better sensor functionality to 80 ppm of acetone at 300 °C for the sensors based on *W* and *Ce*/*W* films and 400 °C for the sensors based on *Ce* films. As the *Ce* based sensors registered comparatively higher baseline resistances (~40 GΩ at 400 °C) than *W* (13 kΩ at 300 °C) and *Ce*/*W* (65 kΩ at 300 °C) sensors, additionally requiring higher temperatures to achieve the maximum responses (e.g., response to acetone 4.9 at 400 °C for the *Ce* sensors and 5.6 at 300 °C for the *Ce*/*W* sensors), further analyses related to the analyte concentration and humidity dependence of the sensor response were performed only for the *W* and *Ce*/*W* based sensors at 300 °C. 

**Figure 7:**
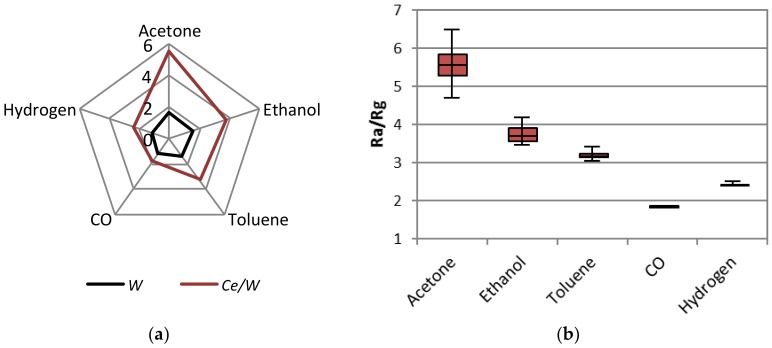
(**a**) The radial plot of the sensor response to 80 ppm of acetone, ethanol, toluene, carbon monoxide, and hydrogen using the *W* and *Ce*/*W* based sensors. (**b**) Box plots of the sensor response to each analyte recorded by the *Ce*/*W* based sensors. Each box displays the median and upper and lower quartiles (first and third) of the respective distribution. Box whiskers indicate the dispersion of the measurements.

**Figure 8:**
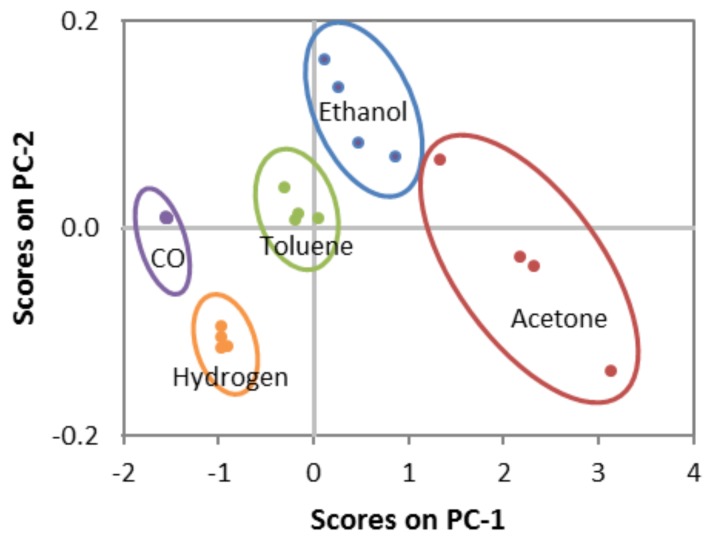
Principal component analysis applied to discriminate the tested VOCs by using an array of non-modified tungsten oxide wires and the cerium oxide-tungsten oxide core-shell wire-based sensors.

Figure 7a displays the sensor response to 80 ppm of each analyte and type of sensor at 300 °C. These results show the improved responses registered for the *Ce*/*W* sensors, as opposed to the *W* sensors, as well as the higher responses to acetone compared to the rest of the analytes. Results in Figure 7a also suggests an improved selectivity for the *Ce*/*W* films with lower cross-responses among the analytes; for instance, the difference of the response to acetone in relation to ethanol is 1.6 for the *Ce*/*W* films and 1 for the *W* films. The low cross response registered on the *Ce*/*W* sensor is noticed in more detail in Figure 7b, in which is displayed the analysis of variance (ANOVA) realized for a data set comprising four replicates for each type of sensor and analyte. Additionally, the principal component (PC) analysis performed using replicated responses of both sensors (i.e., *W* and *Ce*/*W*) to each analyte is represented in Figure 8. These results, specifically the scores, which correspond to the projections of the measurements in an orthogonal base of PCs, indicate the possibility to improve the discrimination of the analytes by using an array of *W* and *Ce*/*W* based sensors. 

**Figure 9:**
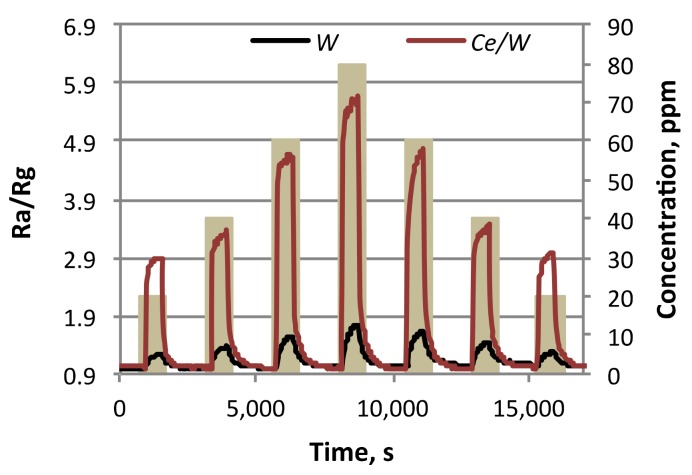
The sensor response to various concentrations (from 20 to 80 ppm) of acetone recorded with the *W* and *Ce*/*W* based sensors.

Further tests of the sensors to various concentrations of each analyte showed direct proportional changes in the response to concentration. An example of the response registered with both types of sensors to acetone is shown in Figure 9. For these conditions, the limit of detection corresponding to three times the noise level [25] was estimated at 1 ppm for *W* and 0.2 ppm for *Ce*/*W* sensors. Overall, the changes respect to concentration for the *W* sensors proved a lower sensitivity compared to the *W/Ce* sensors, which demonstrates a better sensitivity to the analytes. The sensitivity (S), defined as the ratio between the change in response (ΔR) and a fixed change in analyte concentration (ΔC) for each sensor and analyte, was registered to be nearly five times higher for acetone and three times higher for ethanol when using the cerium-modified sensors (found ΔR/ΔC_acetone_ for *W* sensors 0.8%, *Ce*/*W* sensors 4.7%; ΔR/ΔC_ethanol_ for *W* sensors 0.8%, *Ce*/*W* sensors 2.2%). 

**Figure 10:**
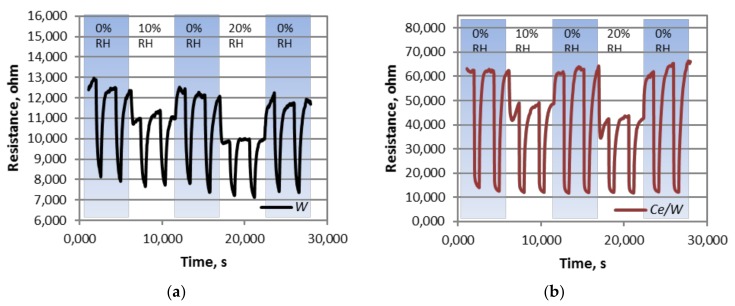
The resistance changes registered for (**a**) *W* and (**b**) *Ce*/*W* to acetone in dry and humid ambient (RH: relative humidity).

Additional tests of the sensors in a controlled humid ambient (10 and 20% RH), consistent with those reported after preconditioning the relative humidity in breath samples [26], registered lower sensor response to the analytes. The loss of response in humid ambient is a consequence of the proportional drop of the baseline resistance to relative humidity. This proportional change is usually present in metal oxides exposed to humidity due to the formation of hydroxyl groups at temperatures above 100 °C [18]. Currently, most of the strategies to attenuate further the humidity interference from the material point of view are connected with the fine tune of the MOX morphology [27] and/or the incorporation of humidity-insensitive additives (e.g., NiO [28], CuO [12], or SiO_2_ [29]). Figure 10 displays the typical resistance changes for the *W* and *Ce*/*W* sensors to each mixture of RH and acetone tested and their replicates. 

Previous reports in the literature related to acetone sensing using modified tungsten oxide films (with Au, Pd, AuPd) suggest the functionality of tungsten oxide at 300 °C for relatively high acetone concentrations (200–1000 ppm) [30]. Further tests, also performed on tungsten oxide, modified with TiO_2_ [31] or Si [32] show the functionality of the sensors at similar (30 ppm) or lower (100–600 ppb) acetone concentrations, respectively, although requiring higher operating temperatures (400–500 °C), than those needed in this work. On the other hand, the use of cerium oxide as the gas sensitive element has been rarely reported in the literature, with the performance of this material having indicated the potential for sensing VOCs including acetone [33,34]. In general, the responses of the above mentioned non-miniaturized acetone sensors in the literature [30,31,32,33,34] are in the same order of magnitude than our micromachined sensors based on the cerium oxide-tungsten oxide core-shell wires, which suggests the viability of sensor miniaturization without losing the sensitivity of the system when optimizing the sensitive material. In addition, the good reproducibility of the responses during the testing period and the analysis (SEM, XRD) of the samples after the gas sensing experiments (which showed unchanged properties of the material with respect to the properties recorded initially) indicated a good stability of the sensors.

**Figure 11:**
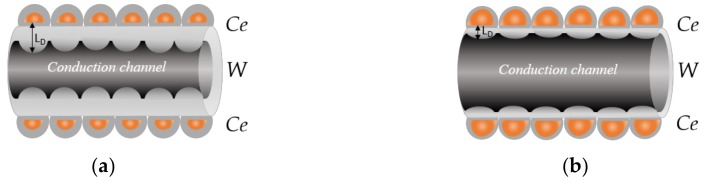
The schematic view of the heterojunction formed at the surface of the cerium oxide (*Ce*) core-shell tungsten oxide (*W*) wires and the possible mechanism **(a)** after exposure to air and **(b)** reducing gases such as acetone. L_D_ is the Debye length or depth of the depletion region from the surface (not at scale).

The enhanced functionality recorded on the *Ce*/*W* sensors is connected with the formation of heterojunctions at the interface of the tungsten oxide wires and the cerium oxide porous films. These heterojunctions are present due to the different band energies in both MOXs (Figure 6b), which facilitate electron migration from the cerium oxide film to the tungsten oxide wire. Generally, as oxygen is preadsorbed at the sensitive film during air exposure, the surface depletion region (L_D_) and, in turn, the conduction channel along the wires are narrowed, which leads to lower conductivity along the film (Figure 11a). Alternatively, when a reducing analyte (i.e., VOCs) reacts with the preadsorbed oxygen, electrons are released back to the conduction band, and the depth of the surface depletion is narrowed, increasing the conduction channel along the wire and, in turn, its conductivity (Figure 11b). This mechanism controlled by the pre-adsorbed oxygen is similar for the non-modified and modified films, with the peculiarity that the charge transfer process (electron migration) occurring at the junction of the cerium oxide film and tungsten oxide wire provides larger electron density to the wires (accumulation layer) in the pre-adsorption cycle (as opposed to the non-modified *W* or *Ce* films). This allows for larger changes of the depletion layer and an enhanced modulation of the wire conduction channel, which is reflected finally on the sensor response. 

In the same line, the lowering of the baseline resistance upon humidity implies a diminution of the chemically active oxygen species at the surface and, in turn, a narrow surface depletion in the air (pre-adsorption). Thus, as the conduction channel in humid ambient is wider than in dry ambient (Figure 11), the conduction changes induced by the reducing gas are less significant, and thus the sensor response is as well. 

Summarizing, the gas sensing tests showed improved acetone sensing properties for *Ce*/*W* based microsensors showing a higher response, and better sensitivity and selectivity to the analytes tested in relation to the *W* or *Ce* based microsensors.

## 4. Conclusions

These results demonstrate the formation of cerium oxide-tungsten oxide core-shell nanowires with improved response and sensitivity to acetone as compared to non-modified tungsten oxide wires or cerium oxide porous films. Tests of these sensors to acetone in humid ambient showed a drop of the responses as a consequence of the lowering of the baseline resistance due to humidity. Principal component analysis of the responses obtained for each analytes using an array the non-modified and modified sensitive films indicated the possibility to enhance the selectivity of the microsensors by improving the discrimination of analytes. The improved sensitivity is attributed to the formation of heterojunctions at the interface of both oxides (i.e., tungsten oxide and cerium oxide) which leads to an ‘extra’ chemical and electronic sensitization to the modified films as compared to the non-modified films.
